# Dietary Fatty Acids Differentially Associate with Fasting Versus 2-Hour Glucose Homeostasis: Implications for The Management of Subtypes of Prediabetes

**DOI:** 10.1371/journal.pone.0150148

**Published:** 2016-03-21

**Authors:** Nicola Guess, Leigh Perreault, Anna Kerege, Allison Strauss, Bryan C. Bergman

**Affiliations:** 1 Department of Medicine, Imperial College London, Du Cane Road, London, United Kingdom; 2 Diabetes and Nutritional Sciences Division, Kings College London, London, United Kingdom; 3 Division of Endocrinology, Metabolism, and Diabetes, University of Colorado Anschutz Medical Center, Aurora, Colorado, United States of America; Baylor College of Medicine, UNITED STATES

## Abstract

Over-nutrition has fuelled the global epidemic of type 2 diabetes, but the role of individual macronutrients to the diabetogenic process is not well delineated. We aimed to examine the impact of dietary fatty acid intake on fasting and 2-hour plasma glucose concentrations, as well as tissue-specific insulin action governing each. Normoglycemic controls (n = 15), athletes (n = 14), and obese (n = 23), as well as people with prediabetes (n = 10) and type 2 diabetes (n = 11), were queried about their habitual diet using a Food Frequency Questionnaire. All subjects were screened by an oral glucose tolerance test (OGTT) and studied using the hyperinsulinemic/euglycemic clamp with infusion of 6,6^2^H_2_-glucose. Multiple regression was performed to examine relationships between dietary fat intake and 1) fasting plasma glucose, 2) % suppression of endogenous glucose production, 3) 2-hour post-OGTT plasma glucose, and 4) skeletal muscle insulin sensitivity (glucose rate of disappearance (Rd) and non-oxidative glucose disposal (NOGD)). The %kcal from saturated fat (SFA) was positively associated with fasting (β = 0.303, P = 0.018) and 2-hour plasma glucose (β = 0.415, P<0.001), and negatively related to % suppression of hepatic glucose production (β = -0.245, P = 0.049), clamp Rd (β = -0.256, P = 0.001) and NOGD (β = -0.257, P = 0.001). The %kcal from trans fat was also negatively related to clamp Rd (β = -0.209, P = 0.008) and NOGD (β = -0.210, P = 0.008). In contrast, the %kcal from polyunsaturated fat (PUFA) was negatively associated with 2-hour glucose levels (β = -0.383, P = 0.001), and positively related to Rd (β = 0.253, P = 0.007) and NOGD (β = 0.246, P = 0.008). Dietary advice to prevent diabetes should consider the underlying pathophysiology of the prediabetic state.

## Introduction

Overnutrition has fuelled the type 2 diabetes (T2D) epidemic and weight loss remains the primary recourse for preventing the progression from prediabetes to T2D [1]. However, successful weight loss maintenance for most people requires considerable support [1], is notoriously difficult to achieve [1] and may not even be effective at preventing T2D in all subjects [2]. Therefore, refining these interventions to increase their effectiveness, or even identify approaches that lower glucose independent of weight, would be indispensable from a public health perspective.

Dietary fats are of particular interest, as they are known to affect insulin sensitivity via a number of mechanisms [3] and have been linked to T2D incidence in several epidemiological trials [3]. However, the data are inconsistent. For example, monounsaturated fat (MUFA) has been shown to be protective in some studies [4–5] but neutral [6–7] in others. Likewise, where a reduction in saturated fat (SFA) has been found protective in some epidemiologic studies [6,8], interventional studies have failed to confirm the strength of the association [9,10]. Nevertheless, it should be pointed out that these studies largely relied upon T2D incidence as an end-point and did not consider the distinct trajectories of T2D development.

While commonly used as an umbrella term to describe the hyperglycaemic state prior to development of T2D, “prediabetes” in fact describes three separate conditions: isolated impaired fasting glucose (IFG), isolated impaired glucose tolerance (IGT) and IFG±IGT, each with its own distinct pathophysiology [11]. For example, differences in beta cell function have been repeatedly observed [11,12], rendering isolated fasting hyperglycemia and normal 2-hour plasma glucose (2hPG) in people with IFG and the converse in those with isolated IGT[11]. Differences in tissue-specific insulin action have also been described [11,12]. Subjects with IFG demonstrate elevated endogenous glucose production (EGP); while skeletal muscle, but not liver insulin resistance, is implicated in IGT. Altogether, an increasing body of evidence supports the concept of distinct pre-diabetic states, each with its own underlying biologic defects and trajectory in the development of T2D [13].

Therefore, it is possible that the inconsistency in the data relating diabetes risk to specific macronutrients simply represents the heterogeneity by which people develop T2D. For example, the polyunsaturated fat (PUFA) content of skeletal muscle phospholipids has been linked to peripheral insulin sensitivity [14], whereas both MUFA and PUFA appear to have beneficial effects on hepatic insulin sensitivity, the latter of which is antagonized by SFA [15,16]. Together, these observations support the notion that fatty acids may have tissue-specific effects on metabolism. Therefore, the objective of the current analysis was to examine the relationship between dietary fatty acid intake on measures of fasting and 2-hour glucose homeostasis, as well as the mechanisms that underlie each.

## Subjects & Methods

### Subjects

The following subjects were included in the analysis to span a wide range of insulin sensitivity: normoglycemic endurance-trained athletes (n = 14), sedentary lean subjects (body mass index (BMI) <25 kg/m^2^; n = 14), and obese controls (BMI 30–40 kg/m^2^; normoglycemic n = 22), and subjects with prediabetes (n = 12) or type 2 diabetes (T2D; n = 11). All subjects reported <3 hours activity per week, except the athletes. Normoglycemia, prediabetes and T2D were confirmed by oral glucose tolerance testing (OGTT) using definitions according to the American Diabetes Association [11]. All subjects were deemed healthy by medical history, physical exam and screening blood tests. Exclusion criteria included: medications that could affect lipid metabolism (except statins in T2D), thyroid disease, a history of lung disease, pregnancy and severe plasma lipid disorders. Use of glucose-lowering medications excluded all but T2D. Nevertheless, any glucose-lowering medications in the T2D group were suspended for two weeks prior to screening and throughout testing. The study was approved by the Colorado Multiple Institutional Review Board prior to commencement. Participants gave their informed consent according to the principles outlined in the Declaration of Helsinki, including providing written consent with a signed consent form which was previously approved by the Colorado Multiple Institutional Review Board.

### Dietary Questionnaire

Participants completed the National Cancer Institute (NCI) Diet History Questionnaire II (DHQ II) at the screening visit as a comprehensive assessment of baseline habitual diet. The DHQ is a Food Frequency Questionnaire (FFQ) developed to reflect food intake over the most recent 3-month period, validated for the intake of saturated fat (SFA), monounsaturated fat (MUFA), polyunsaturated fat (PUFA) and total energy intake [17]. FFQs have been used frequently in intervention and small cross-sectional studies [18,19]. Further validation was provided by analysing the C18:2 composition of red blood cells, as this PUFA is a commonly used and robust biomarker for PUFA intake [20]. In order to identify underreporting, Goldberg’s equations for calculating the upper and lower cut-offs for energy intake were used [21]. The following coefficients of variations (CV) were used in the equations: within-subject variation in energy intake (CVwEI): 19.8% for women and 18.6% for men; variation between estimated BMR and measured BMR (CVwB): 8.5% and total variation in PAL (CVtP): 15%. The CVs used were taken from the published values from comparable populations [21]. To calculate expected energy expenditure the Schofield equation with an activity factor (PAL) of 2.2 was used for athletes, whereas for all others the Mifflin St-Jeor equation was used with a PAL of 1.55 [21]. In total, 12/85 (14%) subjects underreported and their data were excluded from the analysis.

### Red Blood Cell Fatty Acid Composition

Red blood cell (RBC) membrane composition was performed as previously described [22]. Briefly, RBC’s were washed, homogenized, and lipids extracted as described by Folch [23]. The lipid fraction was dried, resuspended in cold acetone, centrifuged, and the supernatant discarded. Phospholipids in the pellet were transmethylated using Na-methoxide, and extracted in hexane. Concentration and composition analysis of resulting fatty acid methyl esters was performed on an HP 6890 GC with a 30m DB-23 capillary column, connected to a HP 5973 MS.

### Metabolic Studies

Subjects reported to the University of Colorado Clinical Translational Research Center (CTRC) the evening prior to the study to ensure compliance with the overnight fast. The morning of the study day, an intravenous catheter was placed in an antecubital vein for infusions and a sampling catheter was placed in a dorsal hand vein of the contralateral arm. For all blood samples, the heated-hand technique was used to arterialize the blood. Thirty minutes after sampling catheters had been placed, baseline samples were taken for concentration of plasma glucose and insulin, as well as glucose enrichment. Then, a primed (0.03mg/kg), constant infusion (0.04mg/kg/min) of 6,6^2^H_2_-glucose began and continued through the end of the study. After 2-hours of isotope infusion, a hyperinsulinemic/euglycemic clamp commenced with a descending insulin prime followed by a constant infusion of insulin at 40 mU/m^2^/min, according to the methods of DeFronzo et al [24]. Plasma glucose concentration was measured every 5 minutes for 3 hours using a bedside glucose analyser (Yellow Springs Instruments; Yellow Springs, OH) and plasma glucose maintained at ~90mg/dl with a variable dextrose infusion. Dextrose infused to maintain euglycemia was “spiked” with [6,6^2^H_2_-glucose] to minimize changes in isotopic enrichment. Blood samples were taken over the final 30 minutes for concentration of plasma glucose and insulin, and glucose enrichment. Determination of resting metabolic rate was made for 15 minutes during the final 60 minutes of the clamp using indirect calorimetry.

### Blood Sample Analyses

All samples were frozen at -80°C until analysis. Radioimmunoassay was used to determine insulin concentration (Linco Research Inc., St. Louis, MO). Standard enzymatic assay was used to measure glucose (COBA-Mira Plus; Roche Diagnostics; Mannheim, Germany).

### Isotope Analysis

Glucose isotopic enrichment was measured using gas chromatography/mass spectrometry (GCMS; GC model 6890 and MS model 5973A, Hewlett-Packard) using standard techniques [25].

### Calculations

Glucose rate of appearance (Ra) and disappearance (Rd) were calculated according to Steele, modified for stable isotopes as previously described for basal conditions [26], as well as during the insulin clamp [27] Glucose Ra and its suppressability with insulin were used to estimate hepatic insulin sensitivity, whereas glucose Rd was normalized to plasma insulin concentration and used as a measure of whole body insulin sensitivity in conjunction with non-oxidative glucose disposal (NOGD). NOGD was calculated as the difference between glucose Rd and carbohydrate oxidation (measured by indirect calorimetry), using standard equations.

### Statistical Analyses

The DHQ II was analyzed using the NCI Diet*Calc software (Bethesda, MD). All nutrient variables were energy adjusted in order to control for the potential confounding effect of total energy intake. Data for specific fatty acids and other macronutrients are presented, as %kcal from that fatty acid or macronutrient. Normality tests were performed for all variables and non-normal variables were transformed prior to analysis. Comparisons between the control, athletes, obese, prediabetes and T2D groups were performed by ANOVA (Tukey; Welch, Dunnett's). To inform regression analyses and identify potential colinearity, a correlation matrix was constructed for nutrient and physiologic variables. Associations between dietary factors and measures of glycaemic outcomes were studied by multiple regression with FPG, % suppression of hepatic glucose production (%HGP), 2hPG, clamp Rd and non-oxidative glucose production (NOGD) as dependent variables. All regression analyses were adjusted for BMI, age, and % kcal from SFA, PUFA and MUFA. Standardised β values are reported to enable comparison of effect sizes between dietary fats, BMI and age. Statistical analyses were performed using the SPSS statistical software for Windows, version 14.0 (SPSS, Chicago, IL). A p-value of <0.05 was considered as significant.

## Results

### Subjects

Subject characteristics are shown in Table 1. Athletes, control and obese subjects were significantly younger than people with T2D or prediabetes (P<0.05 for all comparisons). By study design, subjects in the obese, prediabetes and T2D groups weighed more and had a significantly higher BMI than controls and athletes, but there were no differences in BMI or weight between the obese, prediabetes and T2D groups. As expected, glucose regulatory parameters (i.e. plasma glucose concentrations, glucose Rd and NOGD) for subjects with T2D or prediabetes were significantly different from the other groups (P<0.001 for all comparisons). The T2D group had significantly higher FPG (P<0.001) and 2hPG (P<0.001) than the prediabetes group, but the glucose regulatory parameters were not different (Table 1). Habitual dietary intake is also outlined in Table 1. Red blood cell C18:2 composition was significantly correlated with reported C18:2 intake (r = 0.302, p = 0.024) (S1 Fig).

**Table 1:** Baseline characteristics of study subjects including measures of glucose homeostasis and dietary intake.

	Mean ±± SEM				
	Athlete (n = 14)	Control (n = 14)	Obese (n = 22)	Prediabetes (n = 12)	T2D (n = 11)
Gender (M:F)	9:5	7:7	12:10	2:10	6:5
Age (years)	42.8 ±± 1.4^a^	41.1 ±± 1.6^a^	39.1 ± 1.3^a^	47.0 ± 2.3	46.9 ± 2.0
Weight (kg)	72.4 ±± 2.8	65.0 ±± 2.9	110.3 ± 4.2^b^	96.9 ± 4.3^c^	100.0 ± 6.3^b^
BMI (kg/m^2^)	23.1 ±± 0.6	22.3 ±± 0.7	35.8 ± 4.2^b^	36.0 ± 1.5^b^	34.9 ± 1.8^b^
FPG (mg/dL)	84.7 ±± 1.5^d^	87.0 ±± 2.7^d^	90.1 ± 1.8^d^	103.2 ± 4.4^e^	153.2 ± 10.9
2hPG (mg/dL)	71.0 ±± 5.4^d^	92.3 ±± 4.6^d^	99.6 ± 5.2^d^	102.1 ± 9.0^e^	280.6 ± 19.8
Insulin (μU/ml)	7.0 ±± 2.4^d^	8.9 ±± 1.3^d^	18.7 ± 8.6	19.5 ± 3.7	21.2 ± 9.8
Basal Ra (mg/kg/min)	2.0 ±± 0.0	1.9 ±± 0.1^e^	1.5 ± 0.1^e^^f^*	1.8 ± 0.1^e^	2.4 ± 0.1
HGP (% Ra suppression)	93.2 ±± 2.5^g^	95.2 ±± 2.3^g^^h^	78.3 ± 6.4^*^	50.8 ± 8.1	76.0 ± 4.3
Clamp Rd (mg/kg/min)	12.1 ±± 0.7	8.9 ±± 0.8^c^	4.7 ± 0.5^c^*	4.4 ± 0.5^c^	2.6 ± 0.4^c^
Clamp GIR (mg/kg/min)	11.9 ±± 0.8	8.90 ±± 0.8^c^	4.4 ± 0.5^c^*	3.5 ± 0.5^c^	2.1 ± 0.5^c^
NOGD (mg/kg/min)	12.0 ±± 0.7	8.9 ±± 0.8	4.7 ± 0.5^c^*	3.7 ± 0.6^c^	2.5 ± 0.4^c^
EI (kcal)	2501 ±± 354	2214 ±± 433	2237 ± 128	1938 ± 110	2449 ± 269
Fat	34.7 ±± 1.4	36.0 ±± 1.7	37.1 ± 1.3	39.1 ± 1.7	38.3 ± 1.4
SFA	10.0 ±± 0.5	11.0 ±± 1.0	12.7 ± 0.5	12.3 ± 0.5^i^	13.1 ± 0.6
C8:0	0.1 ±± 0.01	0.1 ±± 0.01	0.1 ± 0.01	0.1 ± 0.01	0.1 ± 0.01
C10:0	0.2 ± 0.03	0.2 ± 0.02	0.2 ± 0.02	0.2 ± 0.04	0.2 ± 0.04
C12:0	0.3 ± 0.05	0.3 ± 0.02	0.3 ± 0.03	0.3 ± 0.03	0.3 ± 0.03
C14:0	0.9 ± 0.1	1.1 ± 0.01	1.0 ± 0.01	1.0 ± 0.1	1.3 ± 0.01
C16:0	5.5 ± 0.3	6.1 ±± 0.3	6.6 ± 0.3	6.7 ± 0.3^j^	7.0 ± 0.3
C17.0	<0.1	<0.1	<0.1	<0.1	<0.1
C18:0	2.2 ± 0.2	2.7 ± 0.1	2.9 ± 0.1^k^	3.0 ± 0.1^i^	3.1 ± 0.1^i^
C20.0	<0.1	<0.1	<0.1	<0.1	<0.1
C22.0	<0.1	<0.1	<0.1	<0.1	<0.1
PUFA	7.7 ± 0.3	6.8 ± 0.5	6.6 ± 0.3	6.7 ± 0.3	6.7 ± 0.3
C18:2	7.6 ± 0.4	6.8 ± 0.4	6.6 ± 0.3	6.6 ± 0.3	6.6 ± 0.5
C18:3	0.6 ± 0.1	0.6 ± 0.1	0.7 ± 0.0	0.6 ± 0.0	0.7 ± 0.1
C18:4	<0.1	<0.1	<0.1	<0.1	<0.1
C20:4	0.1 ± 0.0	0.1 ± 0.0	0.1 ± 0.0	0.1 ± 0.0	0.1 ± 0.0
C20.5	<0.1	<0.1	<0.1	<0.1	<0.1
C22.5	<0.1	<0.1	<0.1	<0.1	<0.1
C22.6	<0.1	<0.1	<0.1	<0.1	<0.1
MUFA	13.7 ± 0.7	13.3 ± 0.8	14.0 ± 0.5	15.7 ± 1.1	14.8 ± 0.8
C14.1	<0.1^d^	<0.1	<0.1	<0.1	<0.1
C16:1	<0.1	<0.1	0.1 ± 0.0	0.1 ± 0.0	<0.1
C18.1	<0.1	<0.1	<0.1^j^	<0.1^j^	<0.1
C20.1	<0.1	<0.1	<0.1	<0.1	<0.1
C22:1	0.1 ± 0.0	<0.1	0.1 ± 0.0	<0.1	<0.1
Trans	1.3 ± 0.1	1.8 ± 0.1	1.9 ± 0.1	2.1 ± 0.1^k^	1.8 ± 0.2
N = 3	0.7 ± 0.1	0.7 ± 0.1	0.8 ± 0.1	0.8 ± 0.1	0.8 ± 0.1
CHO	49.9 ± 1.7	47.8 ± 2.0	44.3 ± 1.7	44.8 ± 2.0	44.8 ± 2.0
PRO	16.1 ± 0.6	17.9 ± 2.0	17.6 ± 0.6	16.9 ± 1.0	16.9 ± 1.0
Fiber (g)	30.2 ± 4.7	19.6 ± 3.3	19.9 ± 2.1	20.8 ± 2.8	20.8 ± 2.8

All numbers for dietary intake are shown as percent of kcal unless otherwise noted. Differences between groups were analysed by ANOVA with a Bonferroni adjustment for normal and Kruskal Wallis for non-normal variables. Stepwise differences are indicated as follows:

^a^<prediabetes and T2D (P<0.05)

^b^>than control, athlete (P<0.05)

^c^> control and athlete (P<0.001)

^d^<prediabetes and T2D

^e^<T2D (P<0.001)

^f^<athlete (P<0.001)

^g^>prediabetes (P<0.001)

^h^>T2D (P>0.001)

^i^>athlete (P<0.005)

^j^>athlete (P<0.01)

^k^>athlete (P<0.05)

^*^clamp data was not collected for 2 obese subjects due to iv infiltration of the clamp.

Basal Ra: Basal rate of glucose appearance; BMI = Body Mass Index; CHO = carbohydrate; Clamp GIR: Glucose infusion rate during the clamp; Clamp Rd: Rate of glucose disappearance during the clamp; EI = Energy Intake; FPG: Fasting plasma glucose; EGP = % suppression of endogenous glucose production; MUFA = monounsaturated fat; NOGD = Non oxidative glucose disposal; N = 3: omega 3 polyunsaturated fatty acid; PRO = protein; PUFA = polyunsaturated fat; SFA = saturated fat; trans = trans fat; T2D = Type 2 diabetes; 2hPG = 2-hour plasma glucose.

### Dietary Intake and Plasma Glucose Concentration

To exclude potentially confounding dietary variables, an expanded analysis was conducted to determine the impact of the major macronutrients on the parameters of interest. The % kcal from total fat, carbohydrate or protein did not predict plasma glucose concentration or the relevant regulatory processes. In contrast to the lack of association of % total dietary fat with glucose regulation, a number of specific fatty acid classes, and individual fatty acids were found as significantly related.

### Relationship between Dietary Fat Intake and Measures and Determinants of Fasting Glucose

**Fig 1:**
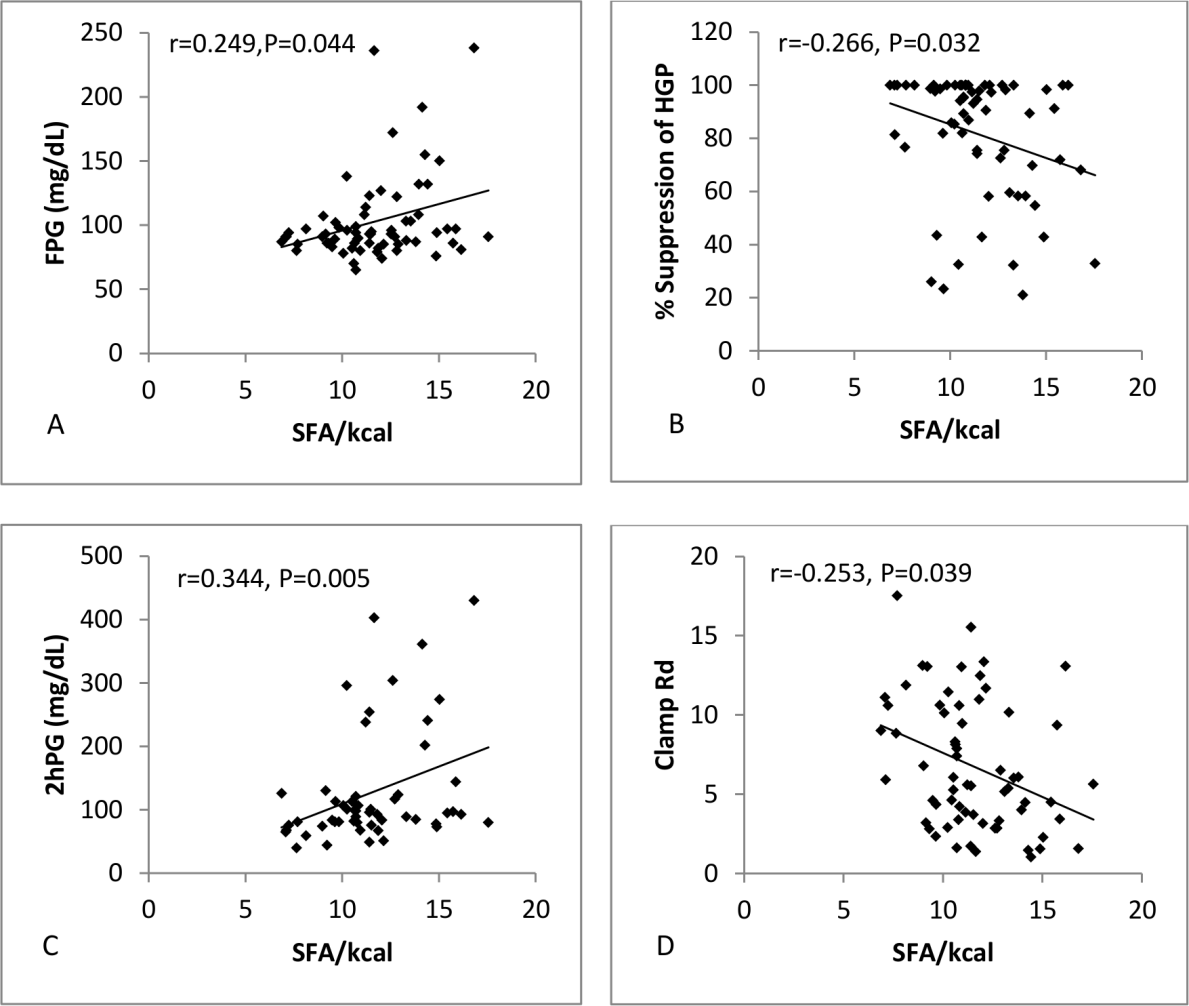
Scatterplots showing %kcal from SFA plotted against (A) FPG (n = 73), (B) % suppression of EGP (n = 71), (C) 2hPG (n = 73) and (D) clamp Rd (n = 71).

**Fig 2:**
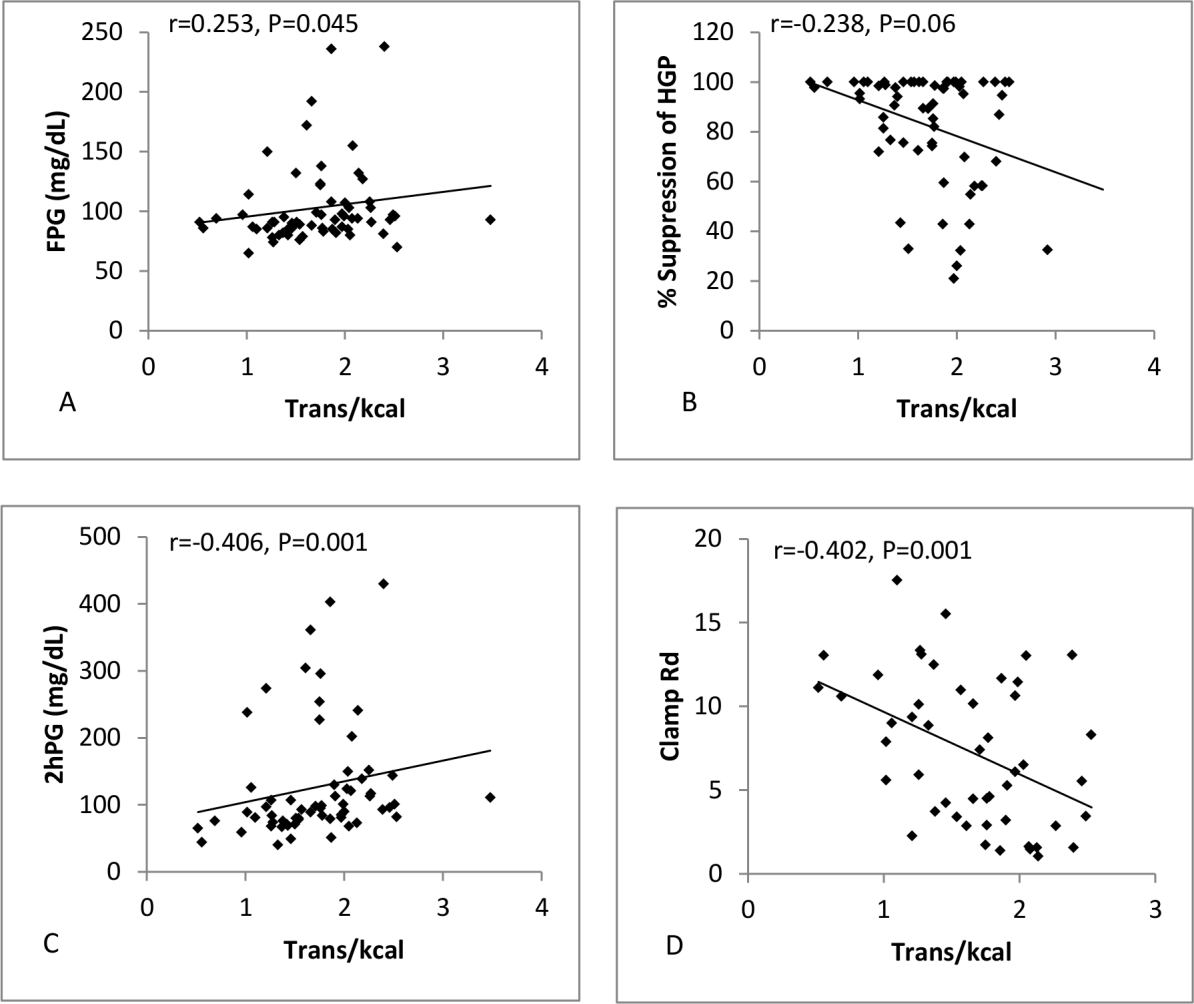
Scatterplots showing %kcal from trans fat plotted against (A) FPG (n = 73), (B) % suppression of EGP (n = 71), (D) 2hPG (n = 73) and (D) clamp Rd (n = 71).

**Fig 3:**
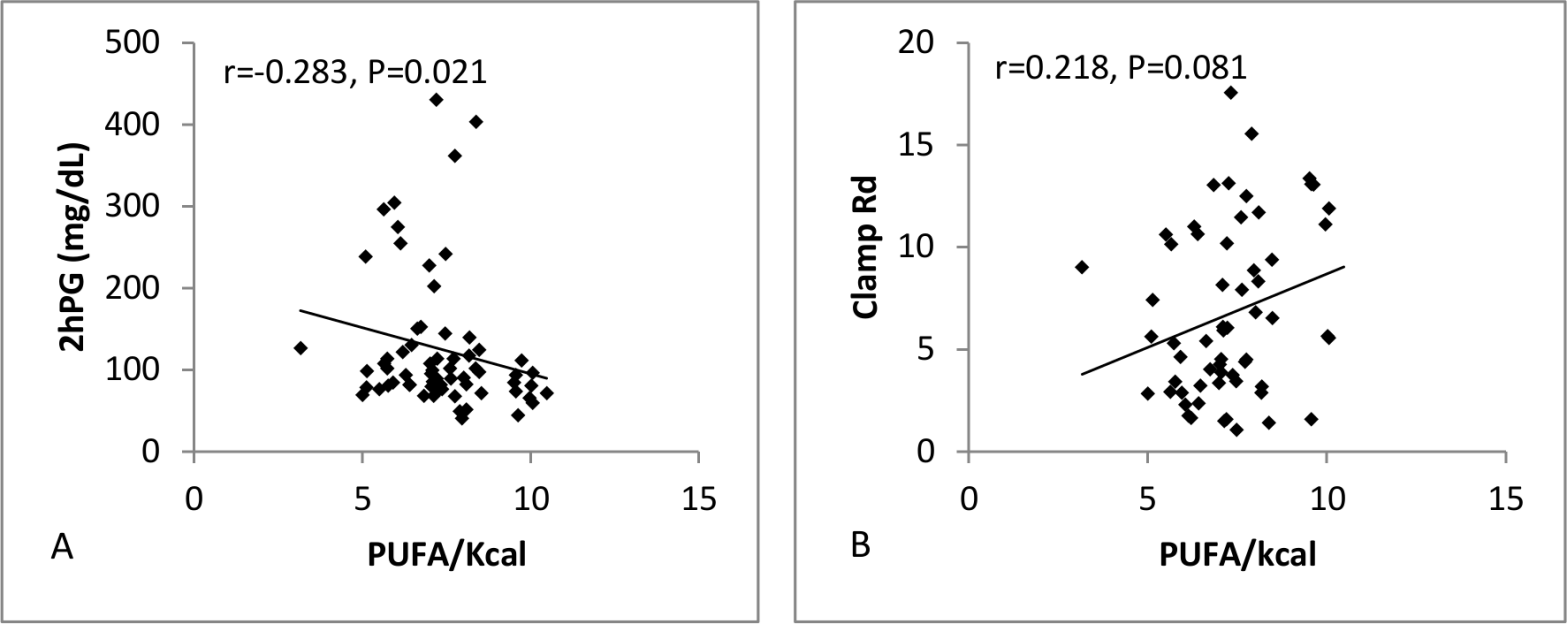
Scatterplots showing %kcal from PUFA plotted against (A) 2hPG (n = 73) and (B) clamp Rd (n = 71).

**Fig 4:**
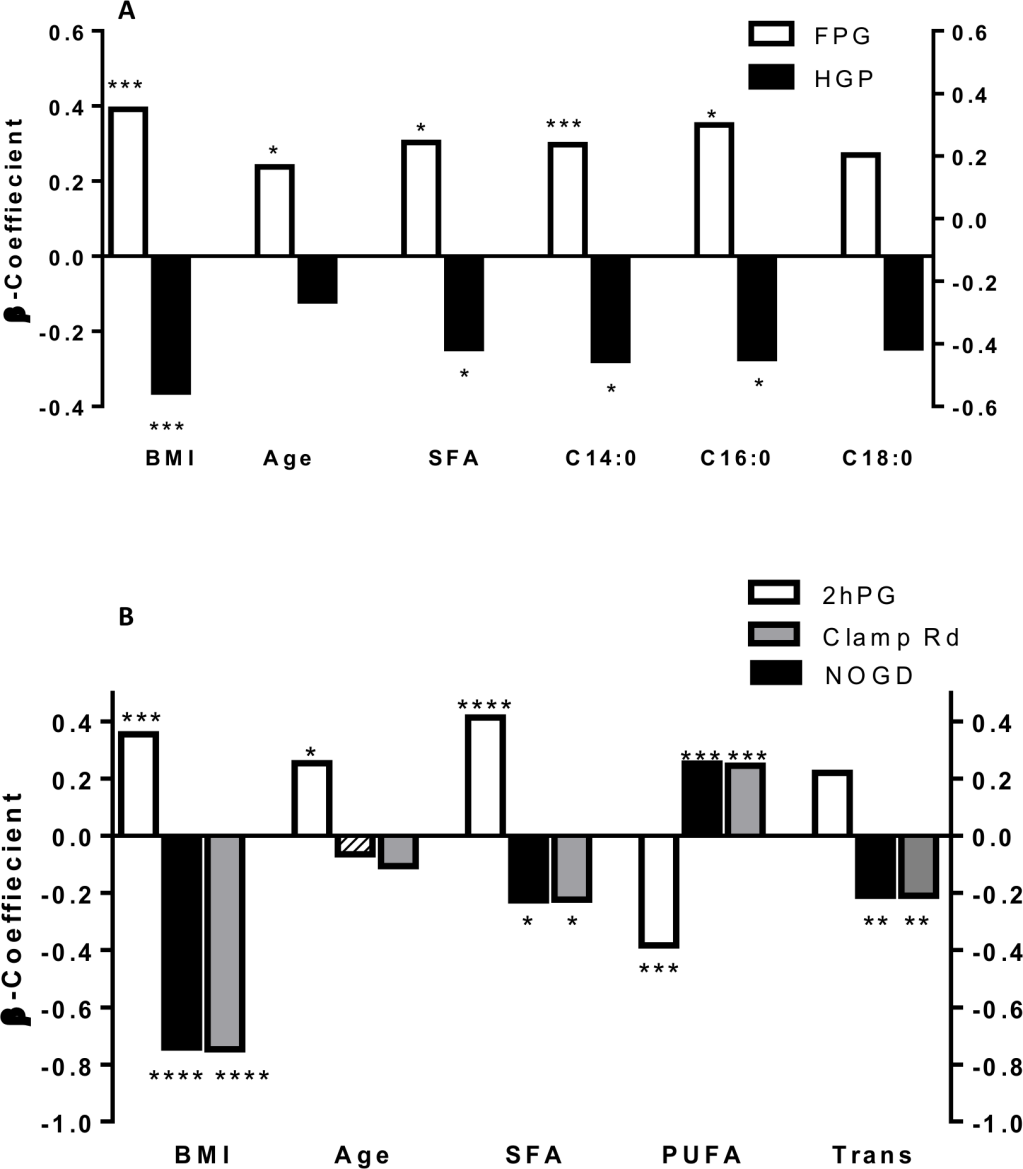
Bar graph illustrating the β-coefficients for A) FPG (n = 73), % suppression of HPG (n = 71) for the following variables: %kcal from SFA, %kcal from C14:0 (myristic acid), C16:0 (palmitic acid) and C18:0 (stearic acid) and B) 2hPG (n = 73), clamp Rd (n = 71) and NOGD (N = 71) for %kcal from SFA, %kcal from PUFA and %kcal from trans fat The β-Coefficients for BMI and age are shown for comparison. Clamp Rd = glucose rate of disappearance; FPG = Fasting Plasma Glucose; EGP = % suppression of endogenous glucose production; NOGD = Non oxidative glucose disposal; 2hPG = 2-hour plasma glucose; * = p<0.05 ** = p<0.01 *** = p<0.001 **** = p<0.0001

Both SFA (Fig 1) and trans fat (Fig 2) were positively associated with FPG (SFA: r = 0.249, P = 0.004; Trans: r = 0.253, P = 0.045) and negatively associated with %HGP (SFA: r = -0.266, P = 0.032; Trans: r = -0.238, P = 0.06), while PUFA (Fig 3) showed no association with either FPG (r = 0.09, P = 0.497) or %HGP (r = -0.101, P = 0.425). To further assess the strength of the relationship between fatty acid intake and fasting plasma glucose (FPG), and to control for potential confounding factors, a multiple regression analysis was performed. The regression model included age, BMI, PUFA, SFA and MUFA. Gender was not related to FPG (P = 0.95) or %HGP (P = 0.33) and so was not included in the regression model. SFA and several specific saturated fatty acids showed a significant positive relationship to FPG (β = 0.303, P = 0.018) and a significant negative correlation with % suppression of HGP with (β = -0.245, P = 0.049) (Figs 1 and 4, S1 Table). As the data for %HGP violated the assumption for linearity even after transformation, we also analysed the data after removing the 20 subjects with 100% suppression of HGP, and found the relationship between SFA and %HGP was strengthened (β = -0.280, P = 0.017) (S2 Table). Following adjustment for BMI, age, and the other dietary fat classes, trans fat was no longer associated with FPG or %HGP. Similarly, total fat, PUFAs (including n-3 fatty acids), MUFAs were neither associated with FPG or % suppression of HGP (S2 Table). Sensitivity analyses were performed in the individual groups and demonstrated consistent directional effects, but non-significant p values due to the small sample sizes. Therefore we analysed and present data for the whole cohort (the five groups together) so that power to determine differences was preserved.

### Relationship between Dietary Fat Intake and Measures and Determinants of 2-hour Plasma Glucose

Both SFA (Fig 1) and trans fat (Fig 2) were positively associated with 2hPG (SFA: r = 0.344, P = 0.005; Trans: r = 0.406, P = 0.001), and negatively associated with clamp Rd (SFA: r = -0.253, P = 0.039; Trans: r = -0.402, P = 0.001). In contrast, PUFA (Fig 3) was negatively associated with 2hPG (r = -0.283, P = 0.021), and positively associated with clamp Rd (r = 0.218, P = 0.081). We again performed multiple regression for 2hPG, clamp Rd and NOGD with adjustment for BMI, age, SFA, PUFA and MUFA but not gender. Overall %kcal from SFA (β = 0.415, P<0.001) (Figs 1 and 4, Table 2), and broadly the same saturated fatty acids (Table 2) which were positively related to FPG, were also positively related to 2hPG, and negatively related to clamp Rd (β = -0.225, P = 0.013) and NOGD (β = -0.223, P = 0.013) (Figs 1 and 4, Table 2). Percent kcal from trans fat was negatively related to clamp Rd (β = -0.209, P = 0.008) and NOGD (β = -0.210, P = 0.008) (Figs 2 and 4, Table 2). In contrast, PUFA was associated with significantly lower 2hPG values (β = -0.383, P = 0.001) and significantly increased clamp Rd (β = 0.253, P = 0.007) and NOGD values (β = 0.246, P = 0.008) (Figs 3 and 4, Table 2). Consistent directional effects were observed when we analysed the data between groups, but again the p values were non-significant due to the small sample sizes. Data is therefore presented for the cohort as a whole.

**Table 2:** Results of multiple regression analyses on parameters of 2hPG homeostasis. The regression model includes age, BMI, SFA, PUFA and MUFA.

	2hPG		Clamp Rd		NOGD	
	β	P Value	β	P Value	β	P Value
**SFA**	0.415	<0.001	-0.225	0.013	-0.223	0.013
**C8:0**	0.301	0.004	-0.07	0.38	-0.074	0.35
**C10:0**	0.319	0.002	-0.121	0.13	-0.127	0.11
**C12:0**	0.131	0.22	0.021	0.79	-0.032	0.68
**C14:0**	0.354	0.001	-0.189	0.016	-0.188	0.015
**C16:0**	0.471	0.001	-0.283	0.005	-0.275	0.005
**C17:0**	0.172	0.17	-0.078	0.38	-0.102	0.24
**C18:0**	0.388	0.003	-0.235	0.017	-0.226	0.02
**C20:0**	-0.259	0.114	0.253	0.022	0.245	0.025
**C22:0**	-0.175	0.215	0.188	0.056	0.193	0.047
**PUFA**	-0.383	0.001	0.253	0.007	0.246	0.008
**C18:2**	-0.380	0.002	0.244	0.009	0.242	0.009
**C18:3**	-0.182	0.11	0.129	0.13	0.132	0.17
**C18.4**	-0.115	0.27	0.115	0.13	0.071	0.95
**C20:4**	-0.238	0.038	0.082	0.32	0.071	0.39
**C20:5**	-0.137	0.19	0.110	0.15	0.082	0.28
**C22:5**	-0.183	0.08	0.127	0.09	0.102	0.17
**C22:6**	-0.184	0.07	0.149	0.047	0.119	0.11
**MUFA**	-0.163	0.95	-0.71	0.47	-0.072	0.46
**C14:1**	0.058	0.59	-0.044	0.59	-0.059	0.47
**C16:1**	-0.08	0.60	-0.102	0.41	-0.068	0.58
**C18:1**	0.006	0.96	-0.072	0.46	-0.071	0.46
**C20:1**	-0.076	0.56	-0.042	0.64	0.035	0.72
**C22:1**	-0.08	0.40	0.095	0.19	0.071	0.33
**n = 3**	-0.044	0.68	-0.133	0.12	-0.159	0.06
**Trans**	0.221	0.08	-0.209	0.008	-0.210	0.008

## Discussion

Dietary recommendations for prevention of diabetes [28] have arisen largely from epidemiological and controlled studies which used diabetes incidence as the primary outcome, and did not take into account whether diabetes developed via FPG or 2hPG [8,9]. Given the distinct pathophysiology of the prediabetic states and the potential tissue-specific effects of dietary fats to render them [12,13], we hypothesized that dietary fat would differentially associate with FPG versus 2hPG glucose homeostasis. Major findings from the current analysis confirm this speculation. Dietary SFA intake corresponded to higher FPG and 2hPG concentration, as well as their physiologic regulators, whereas dietary trans fats and PUFA had opposing effects that were limited to processes regulating 2hPG. Further, self-reported dietary fat intake was corroborated by examining the fatty acid composition of phospholipids in red blood cell membranes [20], dramatically strengthening the objectivity of the results. Together, these data support the notion that diets may be tailored to the subtype of pre-diabetes for the purpose of diabetes prevention.

Our finding that SFA is positively related to FPG supports findings from a number of previous investigations [29–31], including an association between dietary SFA and elevated risk for IFG [30]. Further, our results highlight the robust association of dietary SFA and hepatic insulin resistance, the primary determinant of FPG. And although the current data are cross-sectional in nature, infusion of palm vs. safflower oil in humans under experimental conditions has formerly been shown to selectively increase hepatic glucose output [31]. Hence, one may speculate the same is true for a habitual diet high in SFA. The current analysis would contend that habitual SFA intake is as detrimental for hepatic insulin sensitivity as is increasing BMI, when modelled as independent variables in people spanning a wide range of insulin sensitivity. Taking our data with others, these collectively support the widespread recommendation to reduce dietary SFA in pursuit of optimal metabolic health.

We also observed an association between habitual SFA intake with higher 2hPG, concomitant with a decrement in peripheral insulin action. A reduction in non-oxidative glucose disposal (NOGD) is an important metabolic defect observed in IGT and T2D [32]. We add to the existing literature by demonstrating that dietary SFA is significantly related to impairments in NOGD in subjects across a range of glycaemia. Dietary SFA has been repeatedly shown to impair postprandial glucose homeostasis [33–35]. Our findings corroborate these previous reports and extend them to demonstrate a negative impact of SFA on both FPG and 2hPG, as well as the processes that underlie them. Future studies should examine whether SFA restriction, specifically, proves a useful approach to reverse IFG and/or IGT.

Whereas current guidelines advocate dietary restriction of SFA for cardiometabolic health, they also recommend absolute elimination of trans fats from the diet [36]. Such recommendations have stemmed from epidemiologic, physiologic and basic science experimentation demonstrating their adverse metabolic effects [36]. Our findings further demonstrate that trans fats selectively impair 2hPG and its underlying regulation even after accounting for SFA intake. The effect of trans fats on glucose homeostasis has been suggested to be isomer-specific with the circulating trans isomer of palmitoleic acid from dairy intake reported to protect against diabetes [37]. The majority of trans fatty acid intake in our study came from octadecanoic acid(~3.5g/day), which is both an industrially-produced and dairy source of trans fat [38]. Therefore we cannot determine whether dairy sources of such fats would have the same deleterious association.

In contrast to our findings demonstrating a detrimental association of dietary SFA and trans fats with 2hPG and its regulatory processes, dietary PUFA intake was associated with lower 2hPG and increased muscle insulin sensitivity. Replacement or SFA or trans fat with PUFA reduces diabetes risk in the majority of studies [5,9,39]. Our data suggest that the effects of dietary PUFA on glucose homeostasis are limited to peripheral insulin sensitivity only. There are some data that support this contention. For example, the risk of gestational diabetes, which is characterised by peripheral insulin resistance, is reduced by diets high in PUFAs [40] and an inverse association has been reported between PUFA intake and IGT [41]. Most importantly, physiological studies like ours, which use validated dietary assessment alongside robust biomarkers for fat intake have shown an inverse relationship between PUFA consumption and peripheral insulin resistance [14,42], while the beneficial effect of dietary PUFA on 2hPG is not observed with respect to lowering FPG [14, 31]. This raises the promising question that targeted dietary interventions may impede or alter the manner in which people develop diabetes. We suggest an exploration of long-term prospective data allowing the analysis of dietary variables on the development of IFG versus IGT.

There are considerable strengths to this study including the use of robust methodology to measure tissue specific glucose homeostasis in a cohort of subjects spanning a wide range of insulin sensitivity. Furthermore, while self-reported data–even for validated questionnaires—has some shortcomings, the corroboration of PUFA intake with RBC data considerably strengthens the objectivity of our results. However, we also acknowledge some limitations. Although all participants in the lean control, obese, prediabetes and diabetes groups reported doing less than 3 hours physical activity per week, we did not objectively measure physical activity, and therefore cannot exclude the possibility that this was a confounding factor in this study. Future research should utilise accelerometers or VO_2_ max. A two-step clamp would have enabled more accurate assessment of HGP suppressibility. Nevertheless, the relationship between SFA and %HGP remained even when those with 100% suppressibility of HGP were removed, suggesting that our use of a one-stage clamp did not alter the finding. We acknowledge the small sample size in this study. In particular, the small number of participants in each group meant that we could not evaluate the relationship between dietary fat and glycaemic outcomes in the sub-groups in any meaningful way. While the direction of the relationship was the same in each group, further studies may consider this point. Finally, the cross-sectional design of the study means we cannot infer causality, and our data were not adjusted for multiple comparisons and are intended to be hypothesis-generating.

## Conclusion

In summary, major findings from the current study demonstrate that dietary SFA intake corresponded to higher fasting and 2hPG concentration, whereas dietary trans fats and PUFA had opposing effects that were limited to processes regulating 2hPG. Our findings raise the possibility that dietary advice targeted to the specific pathophysiological defects in both IFG and IGT could increase the effectiveness of traditional lifestyle modification programs.

## Supporting Information

S1 Fig C18:2 composition of red blood cells plotted against reported C18:2 intake from the National Cancer Institute Food Frequency Questionnaire. (DOCX)

S1 Table Results of multiple regression analyses on parameters of FPG glucose homeostasis. The regression model includes age, BMI, SFA, PUFA and MUFA.
(DOCX)

S2 Table Results of multiple regression analyses on parameters of % suppression of endogenous glucose production, with 20 subjects with 100% suppression of EGP removed to ensure linearity. The regression model includes age, BMI, SFA, PUFA and MUFA.
(DOCX)
