# Supplementary material for: Dietary Fatty Acids Differentially Associate with Fasting Versus 2-Hour Glucose Homeostasis: Implications for The Management of Subtypes of Prediabetes
Source: PLoS One. 2016 Mar 21;11(3):e0150148. doi: 10.1371/journal.pone.0150148 (PMC4801380; doi:10.1371/journal.pone.0150148)
Supplement: S1 Fig — (DOCX) [file pone.0150148.s001.docx]

r= 0.302, p=0.024

S1 Fig : C18:2 composition of red blood cells plotted against reported C18:2 intake from the National Cancer Institute Food Frequency Questionnaire.
