# Supplementary material for: Dietary Fatty Acids Differentially Associate with Fasting Versus 2-Hour Glucose Homeostasis: Implications for The Management of Subtypes of Prediabetes
Source: PLoS One. 2016 Mar 21;11(3):e0150148. doi: 10.1371/journal.pone.0150148 (PMC4801380; doi:10.1371/journal.pone.0150148)
Supplement: S1 Table — The regression model includes age, BMI, SFA, PUFA and MUFA. (DOCX) [file pone.0150148.s002.docx]

|  | **FPG** | | **%HGP** | |
| --- | --- | --- | --- | --- |
|  | **β** | **P Value** | **β** | **P Value** |
| **SFA** | 0.303 | 0.018 | -0.245 | 0.049 |
| **C8:0** | 0.286 | 0.009 | -0.153 | 0.19 |
| **C10:0** | 0.255 | 0.021 | -0.199 | 0.08 |
| **C12:0** | 0.134 | 0.23 | -0.338 | 0.003 |
| **C14.0** | 0.297 | 0.008 | -0.277 | 0.017 |
| **C16.0** | 0.350 | 0.019 | -0.272 | 0.033 |
| **C18.0** | 0.270 | 0.05 | -0.244 | 0.06 |
| **C20:0** | 0.042 | 0.73 | 0.201 | 0.11 |
| **C22:0** | 0.253 | 0.046 | 0.210 | 0.10 |
| **PUFA** | -0.075 | 0.55 | 0.039 | 0.34 |
| **C18:2** | 0.06 | 0.93 | -0.015 | 0.90 |
| **C18:3** | 0.04 | 0.76 | -0.016 | 0.90 |
| **C18:4** | -0.147 | 0.19 | -0.008 | 0.94 |
| **C20:4** | -0.279 | 0.021 | -0.031 | 0.80 |
| **C20:5** | -0.138 | 0.22 | -0.027 | 0.81 |
| **C22:5** | -0.184 | 0.10 | -0.056 | 0.62 |
| **C22:6** | -0.208 | 0.06 | -0.034 | 0.79 |
| **MUFA** | -0.064 | 0.67 | -0.047 | 0.76 |
| **C14:1** | 0.121 | 0.31 | 0.045 | 0.72 |
| **C16:1** | -0.027 | 0.87 | -0.018 | 0.93 |
| **C18:1** | -0.51 | 0.72 | -0.06 | 0.69 |
| **C20:1** | -0.005 | 0.97 | -0.001 | 0.99 |
| **C22:1** | -0.107 | 0.13 | 0.114 | 0.31 |
| **n=3** | -0.182 | 0.12 | -0.087 | 0.52 |
| **Trans** | -0.065 | 0.61 | -0.133 | 0.30 |

S1 Table: Results of multiple regression analyses on parameters of FPG glucose homeostasis. The regression model includes age, BMI, SFA, PUFA and MUFA.
