# Supplementary material for: Dietary Fatty Acids Differentially Associate with Fasting Versus 2-Hour Glucose Homeostasis: Implications for The Management of Subtypes of Prediabetes
Source: PLoS One. 2016 Mar 21;11(3):e0150148. doi: 10.1371/journal.pone.0150148 (PMC4801380; doi:10.1371/journal.pone.0150148)
Supplement: S2 Table — The regression model includes age, BMI, SFA, PUFA and MUFA. (DOCX) [file pone.0150148.s003.docx]

|  | **%HGP** | |
| --- | --- | --- |
|  | **β** | **P Value** |
| **SFA** | -0.280 | 0.017 |
| **C8:0** | -0.098 | 0.43 |
| **C10:0** | -0.228 | 0.039 |
| **C12:0** | -0.374 | 0.001 |
| **C14.0** | -0.328 | 0.004 |
| **C16.0** | -0.317 | 0.011 |
| **C18.0** | -0.297 | 0.018 |
| **C20:0** | 0.240 | 0.056 |
| **C22:0** | 0.273 | 0.032 |
| **PUFA** | -0.036 | 0.74 |
| **C18:2** | -0.002 | 0.99 |
| **C18:3** | 0.153 | 0.25 |
| **C18:4** | 0.138 | 0.20 |
| **C20:4** | 0.161 | 0.16 |
| **C20:5** | 0.158 | 0.14 |
| **C22:5** | 0.191 | 0.075 |
| **C22:6** | 0.160 | 0.14 |
| **MUFA** | -0.031 | 0.84 |
| **C14:1** | -0.013 | 0.91 |
| **C16:1** | -0.048 | 0.81 |
| **C18:1** | 0.023 | 0.88 |
| **C20:1** | 0.01 | 0.95 |
| **C22:1** | 0.05 | 0.65 |
| **n=3** | 0.134 | 0.27 |
| **Trans** | 0.157 | 0.19 |

S2 Table: Results of multiple regression analyses on parameters of % suppression of endogenous glucose production, with 20 subjects with 100% suppression of EGP removed to ensure linearity. The regression model includes age, BMI, SFA, PUFA and MUFA.
